# A retrospective comparison of two-dose and three-dose melarsomine, with doxycycline and a macrocyclic lactone, in 1246 shelter dogs

**DOI:** 10.1186/s13071-026-07344-x

**Published:** 2026-08-20

**Authors:** Linda S. Jacobson, Brian A. DiGangi, Maria A. Serrano, Joey Friedman, Mia K. Cuccinello, Hannah M. Garcia, Julie K. Levy

**Affiliations:** 1 Toronto Humane Society, Toronto, ON Canada; 2 EveryPet, Jacksonville, FL USA; 3 Miami-Dade Animal Services, Miami, FL USA; 4 Shelter Medicine Program, University of Florida, Gainesville, FL USA

**Keywords:** *Dirofilaria immitis*, Heartworm disease, Melarsomine, Doxycycline, Macrocyclic lactone, Adulticide therapy, Treatment efficacy, Complications, Outcomes

## Abstract

**Background:**

Doxycycline and a macrocyclic lactone (ML/doxy) are now standard additions to melarsomine treatment protocols. The safety and efficacy of the two-dose melarsomine protocol have not been revisited since these adjuncts were added to treatment guidelines.

**Methods:**

This was a retrospective cohort study comparing two-dose and three-dose melarsomine in dogs with class 1 and 2 heartworm disease, treated at Miami-Dade Animal Services from 2014 to 2024. All dogs received ML/doxy. Outcome measures were efficacy 5–10 months after the last melarsomine injection, survival to 8 weeks post-melarsomine, and post-treatment complications. Efficacy was assessed for different MLs administered at intake.

**Results:**

Denominators for analyses varied on the basis of available data. There were 889 dogs in the two-dose melarsomine group and 357 in the three-dose group. Follow-up antigen tests were negative in 242/252 dogs (96.0%; confidence interval [CI] 92.9–97.8%) in the two-dose group and 24/24 (100%; CI 86.2–100%) in the three-dose group (*P* > 0.9999; not significant [NS]). All-cause mortality was 4/491 (0.8%) in the two-dose group and 4/154 (2.6%) in the three-dose group (*P* = 0.097; NS). Heartworm-related mortality was 1/491 (0.2%) in the two-dose group and remained at 4/154 (2.6%) in the three-dose group (*P* = 0.013). The deaths in the three-dose group were not considered to be related to the specific treatment protocol. Mild post-melarsomine complications occurred in 66/889 (7.4%) dogs in the two-dose group and 13/357 (3.6%) in the three-dose group (*P* = 0.014). Severe complications occurred in 3/889 (0.3%) dogs in the two-dose group and 5/357 (1.4%) in the three-dose group (*P* = 0.047). Two dogs that initially received ivermectin (2/65; 3.1%) and two that received milbemycin (2/35; 5/7%) had a positive follow-up antigen test, compared with none (0/33) for sustained-release moxidectin.

**Conclusions:**

The findings suggest that two-dose melarsomine, with ML/doxy, is a potential alternative to three-dose melarsomine for class 1 and 2 heartworm disease. Moxidectin may be the preferred ML in the two-dose protocol.

**Graphical Abstract:**

**Figure:**
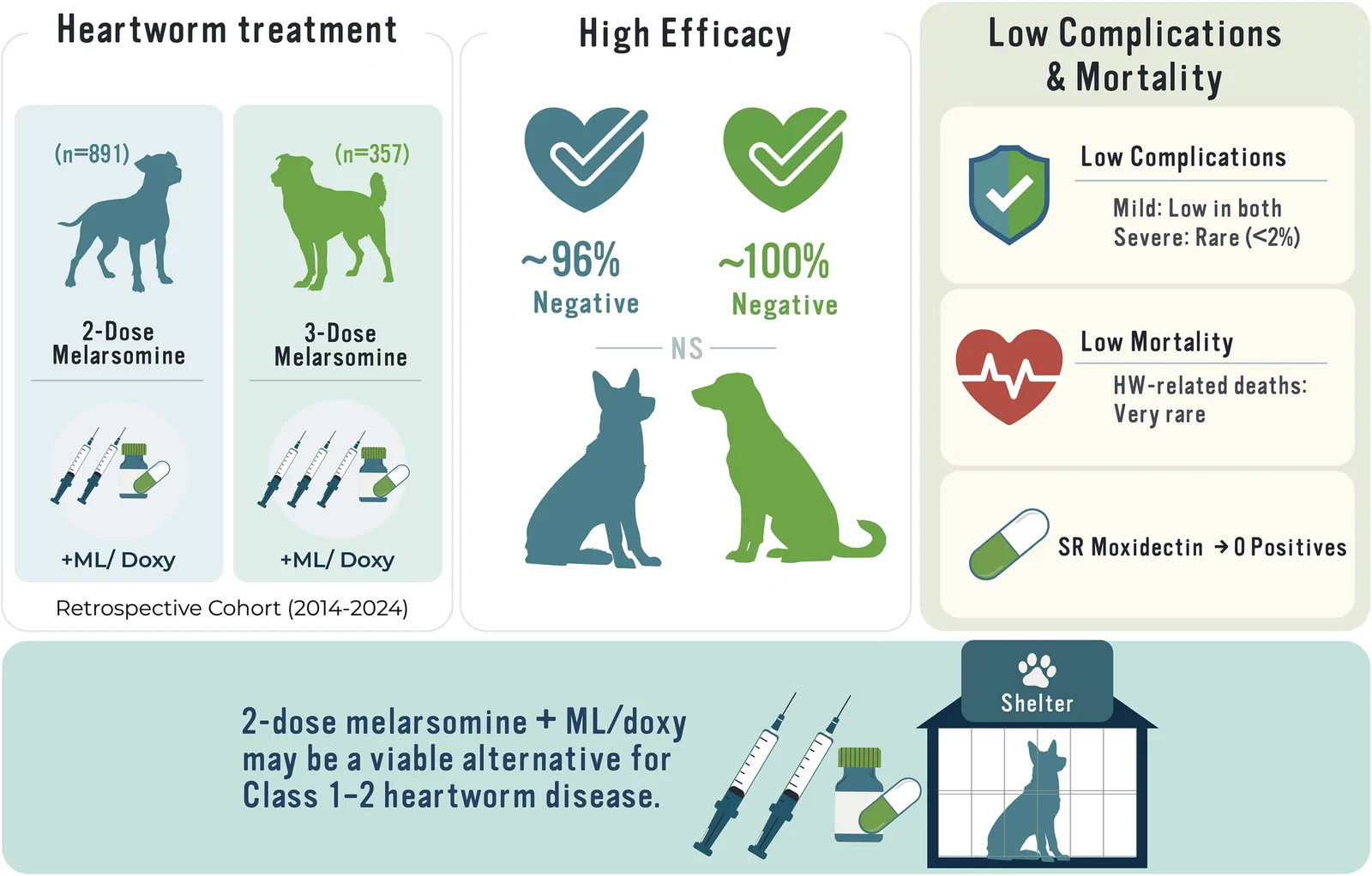

## Background

Drugs derived from arsenic have been used to treat canine heartworm (*Dirofilaria immitis*) infection since the 1950s [1]. Melarsomine was registered in the 1990s and is currently the only Food and Drug Administration (FDA)-approved adulticide treatment for canine heartworm. The American Heartworm Society recommends a three-dose melarsomine protocol consisting of an initial injection 60 days after the initiation of adjunctive treatments, followed by the second and third injections 90 and 91 days after diagnosis [2].

Despite its high safety and efficacy, the three-dose melarsomine protocol requires an investment of veterinary and financial resources [3–5]. Animal shelters have identified cost as the primary challenge to successfully managing canine heartworm disease (HWD) [6]. Cost was also identified as the most important barrier to veterinary care among US pet owners, with 26% reporting that they were able to pay less than US $500 for lifesaving care [7, 8].

Melarsomine has a low safety margin and is highly irritating to tissues [9, 10]. Administration therefore requires close veterinary supervision. The three-dose protocol requires several veterinary visits and sometimes an overnight hospital stay after injections or between the second and third injections. Exercise restriction is recommended from the time of diagnosis until 6–8 weeks after the last melarsomine injection [2], a total of 4.5–5 months. Prior to melarsomine treatment, exercise is restricted proportional to the severity of clinical signs. Strict exercise restriction is recommended after melarsomine injections to reduce the risk of complications from pulmonary thromboembolism (PTE) caused by dead and dying worms [2]. Exercise restriction can be challenging for owners and animal shelters, and may represent a welfare concern for dogs confined to shelter kennels, for whom daily physical activity is an integral part of humane care [11].

The label recommendations for melarsomine [9] are based on early experimental and field studies [12–17] and recommend selecting the treatment protocol according to staging based on clinical, laboratory, and radiographic findings [18]. For class 1 and 2 (asymptomatic to moderate) HWD, the label directions are to administer two injections 24 h apart. The label states that a second series can be considered after 4 months, depending on response to treatment and the condition, age, and use of the dog. For class 3 (severe) HWD, the label calls for a single initial injection, followed by two injections 24 h apart, approximately 1 month later. This kills the worms more gradually and reduces the risk of complications from PTE.

In early experimental studies, the efficacy of two-dose melarsomine was 90.7–100% of worms killed and 50% (3/6) to 100% (3/3) of dogs worm-free [13, 19]. The efficacy of the three-dose protocol was 99% of worms killed and 83.3% (5/6) of dogs worm-free [13]. In early field studies, efficacy (no antigen detected [NAD]) was 70.0–99.2% after two doses [12, 15, 17, 20] and 89.2–98.2% after three doses [12, 14].

Dogs with more severe HWD are at higher risk of post-treatment complications. A staging system was developed in 1992 [18] and has subsequently been adapted to allow classification based primarily on history and clinical signs [2]. In the earlier system, class 1 HWD was defined as subclinical (asymptomatic), with normal to minimal radiographic changes and normal laboratory findings. Class 2 (moderate; now mild to moderate) included mild to moderate clinical signs, moderate radiographic changes, and possibly some laboratory abnormalities. Class 3 (severe) included severe cardiorespiratory signs, marked radiographic changes, and laboratory abnormalities [18]. The safer three-dose, or split, protocol was developed because class 3 dogs had higher post-treatment complications and mortality after treatment with the original two-dose protocol [12, 14, 21]. This is because these dogs are likely to be older, with chronic infections and significant pulmonary pathology. Soon after melarsomine was approved, the three-dose protocol was suggested for all dogs that had not been clinically staged, as 60% of dogs with adverse events were unstaged [22]. The three-dose protocol was later recommended for all dogs. In addition to safety benefits, the rationale was that the higher efficacy reduced the need for repeat treatment and that “staging of the disease and use of the two-injection protocol has failed to adequately ensure treatment success” [23, 24].

Since 2005, adjunctive medications have been included in melarsomine protocols, with the goal of further improving safety by reducing worm biomass and pulmonary inflammation following worm death [23, 24]. One of these adjuncts, the antibiotic doxycycline, reduces pulmonary inflammation and complications from PTE associated with dead and dying heartworms, as well as affecting the worm’s reproduction and ability to thrive [1, 25–32]. This is through its effects on the endosymbiont bacterium *Wolbachia*, which supports the worm’s metabolism and reproduction and releases inflammatory proteins after worm death. Macrocyclic lactone (ML) heartworm preventives, previously recommended only after melarsomine treatment [33], are now initiated at the time of diagnosis to prevent new infections and eliminate immature stages of the parasite [34–36]. Ivermectin has been demonstrated to synergize with doxycycline to weaken and kill all stages of the heartworm parasite [1, 37–41]. Moxidectin and doxycycline were effective against immature adult parasites in an experimental study [42]. Concurrent MLs and doxycycline (ML/doxy) can be used for adulticide treatment independent of melarsomine [43].

The initial studies establishing the safety and efficacy of melarsomine did not include ML/doxy, and no subsequent studies have re-evaluated two-dose melarsomine when used with these adjuncts. The adjuncts have the potential to improve the safety and efficacy of two-dose melarsomine. The two-dose protocol shortens the duration of treatment and exercise restriction by a month. In addition, it reduces the cost of treatment. We hypothesized that the addition of ML/doxy would improve the safety and efficacy of two-dose melarsomine. The purpose of this study was to retrospectively compare two-dose and three-dose melarsomine protocols that included ML/doxy.

## Methods

### Study population and setting

This was a retrospective cohort study of dogs treated for HWD by Miami-Dade Animal Services (MDAS), from February 2014 to July 2024. MDAS is an urban, municipal, open-intake shelter that had an average annual intake of 8000–10,000 dogs during the study period. The shelter houses 400–600 dogs at a time and experiences endemic canine infectious respiratory disease. MDAS operates a full-service licensed veterinary hospital. Case records were retrieved from the shelter database (Chameleon, Chameleon Software Products, Chicago, IL, USA).

### Clinical procedures and protocols

All dogs estimated to be 5 months and older were tested for heartworm (HW) on admission, using EDTA-coagulated whole blood and a point-of-care antigen test (Heartworm SNAP or 4DX SNAP, IDEXX Laboratories, Westbrooke, Maine or WITNESS Heartworm Antigen Test, Zoetis, Parsippany, NJ, USA). If the antigen test was positive, a drop of whole blood was examined microscopically for microfilariae (MF).

HWD was classified by a veterinarian as follows: class 1—no clinical signs; class 2—fatigue upon exercise, occasional coughing; class 3—cardiac cachexia, constant fatigue, persistent cough, dyspnea, heart failure (ascites, jugular pulse, edema). Only dogs with class 1 and 2 HWD were eligible for treatment.

Three treatment protocols were followed during different time periods (Table 1). Two-dose melarsomine was administered during the first and third periods (2014 to mid-2019; 2023–2024), and three-dose melarsomine during the second time period (mid-2019 to late 2022). Doxycycline at 10 mg/kg orally (PO) q 24 h for 28 days was initiated on day 1 of treatment. From 2014 until December 2022, ivermectin or milbemycin were administered on admission and monthly while at the shelter. Sustained-release injectable moxidectin (ProHeart 12, Zoetis, Parsippany, NJ, USA) was administered on admission from January 2023. Prior to January 2023, melarsomine was administered only after adoption. Thereafter, dogs were treated either in-shelter or post-adoption.

**Table 1:** Shelter heartworm treatment protocols during the study period

	Time period 1	Time period 2	Time period 3
Melarsomine injections	2	3	2
Timeframe	February 2014 to July 2019	August 2019 to December 2022	January 2023 to July 2024
*N* dogs	679	357	212
Melarsomine timing	Post-adoption	Post-adoption	In-shelter or post-adoption
At diagnosis	Antigen test, microscopic examination for MF. Heartworm preventive; dexamethasone and diphenhydramine if MF-positive		
Doxycycline^a^	Doxycycline 10 mg/kg q 24 h × 28 days. First day of doxycycline = day 1		
Macrocyclic lactone	Ivermectin or milbemycin	Ivermectin or milbemycin	Sustained release moxidectin
Analgesia for melarsomine	Hydromorphone and dexmedetomidine IM; carprofen SC; gabapentin and trazodone for 10 days		
First melarsomine^b^	Day 60	Day 60	Day 60
Second melarsomine^b^	Day 61	Day 90	Day 61
Third melarsomine^b^	n/a	Day 91	n/a
Exercise restriction	Post-melarsomine, exercise limited to short leash walks for elimination for 6 weeks		

^a^Minocycline was substituted when doxycycline was not available

^b^Timing of injections varied within defined limits, see inclusion criteria

*MF* microfilariae, *ML* macrocyclic lactone, *SC* subcutaneous, *q* every, *IM* intramuscular

### Inclusion criteria

Dogs were considered for inclusion if they had a positive HW antigen test and class 1 or 2 HWD and were excluded if the diagnosis was equivocal (e.g., unresolved dissonant results between two antigen tests). Melarsomine was not always administered at the intervals stated in the shelter protocol (Table 1) because of variable times to adoption, adopter compliance, and shelter resources. Dogs were therefore included if the following criteria were satisfied: ≤ 10 months between the end of doxycycline treatment and the first melarsomine injection; ≤ 24 h between melarsomine treatments intended to be given 24 h apart; ≤ 90 days between the first and second melarsomine injections (three-dose protocol); no more than three melarsomine injections before the repeat antigen test; and melarsomine course completed unless discontinued for a documented medical reason.

### Post-treatment outcomes

Shelter records were searched for post-melarsomine antigen tests performed by the shelter. If the records did not contain results, adopters were contacted by email, text, and/or telephone. Test results were included if they were performed 5–10 months after the last melarsomine injection. This was to include the 6-month timing, previously recommended, and the current 9-month timing, with a month on either side for tests not performed at exactly 6 or 9 months post-melarsomine. Adopter-reported results were confirmed by their veterinary clinics to the extent possible. Survival was defined as survival to ≥ 8 weeks after the final melarsomine injection. Heartworm-related mortality was defined as any death directly attributable to or consistent with HWD or HW treatment. Adverse reactions to medication and post-treatment complications were recorded.

### Data analysis

Analyses were performed according to the intention-to-treat principle, i.e., dogs were analyzed within their intended melarsomine group irrespective of completion. Data were analyzed utilizing Microsoft Excel and GraphPad Prism 10.6.0. The Mann–Whitney *U* test was used to compare non-normally distributed continuous data between groups. Fisher’s exact test was used to compare categorical variables between groups. Significance was set at *P* < 0.05.

## Results

**Fig. 1:**
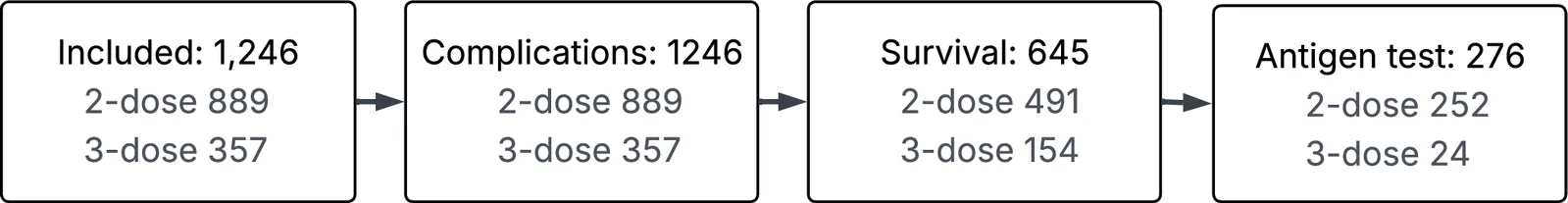
Sample sizes for different study analyses

A total of 1375 dogs met the initial inclusion criteria. Overall, 129 were subsequently excluded, 15 for equivocal diagnosis, 45 for incomplete melarsomine treatment without a documented medical reason, and 69 for protocol violations. Sample sizes for different analyses are shown in Fig. 1.

There were 889 dogs in the two-dose melarsomine group and 357 in the three-dose group. The groups did not differ significantly for age, sex, or weight (Table 2). MF tests were more frequently positive in the two-dose group (44.4%) than the three-dose group (37.2%) (Fisher’s exact test, two-sided *P* = 0.023). The first melarsomine injection was given a median of 71 days (interquartile range [IQR] 62–96) after initiation of doxycycline in the two-dose group and 74 days (IQR 64–112) in the three-dose group (Mann–Whitney *U* = 139,814, two-tailed *P* = 0.001). The median time to the second melarsomine injection for the three-dose group was 34 days (IQR 28–35; range 27–77) (Table 2).

**Table 2:** Baseline data for 1246 dogs administered two-dose or three-dose melarsomine in a shelter setting

	Two-dose	*n*	Three-dose	*n*	*P*-value
All		889		357	
Age (years)	3 (2–4; 0.6–13)	886	3 (2–4; 0.6–10)	348	0.796
Sex		887		356	0.565
FI	301 (33.9%)		136 (38.2%)		
FS	82 (9.2%)		31 (8.7%)		
MI	420 (47.4%)		158 (44.4%)		
MN	84 (9.5%)		31 (8.7%)		
Weight (kg)	24 (18–29; 2.3–73)	889	24 (19–29; 2.7–56)	357	0.54
Positive MFT at intake	380 (44.4%)	855	127 (37.2%)	341	**0.023**
Day 1 doxy to mel 1 (d)	71 (62–96; 15–299)	889	74 (64–112; 31–299)	357	**0.001**
Mel 1 to mel 2 (d)	1	889	34 (28–35; 27–77)	356	–
Mel 2 to mel 3 (d)	n/a	–	1	354	–

Data are shown as median (IQR; range) or *n* (%). Significant *P* values are shown in bold

*FI* intact female, *FS* spayed female, *MI* intact male, *MN* neutered male, *doxy* doxycycline, *Mel 1* first melarsomine injection, *Mel 2* second melarsomine injection, *Mel 3* third melarsomine injection, *d* days

Key outcomes are presented in Table 3. Follow-up antigen tests were negative in 242/252 dogs in the two-dose group (96.0%; CI 92.9–97.8%) and 24/24 (100%; CI 86.2–100%) in the three-dose group (Fisher’s exact test, two-tailed *P* > 0.9999; not significant [NS]). The median time to testing after the final melarsomine injection was significantly shorter for the two-dose group (Mann–Whitney *U* = 2184, two-tailed *P* = 0.014). Of the ten positive tests in the two-dose group, seven were performed 6 months post-melarsomine. None of these tests were repeated at later time points. In 26 cases, an MF test was included with the follow-up antigen test. All follow-up MF tests were negative; 11 of these dogs had had a positive MF test at intake.

**Table 3:** Key outcomes (efficacy and mortality) for dogs treated with two-dose or three-dose melarsomine

	Two-dose	*n*	Three-dose	*n*	*P*-value
Negative antigen test^a^	242 (96.0%; CI 92.9–97.8%)	252	24 (100%; CI 86.2–100%)	24	> 0.9999
Months to antigen test	6 (6–7; 5–10)	252	8 (6–9; 5–10)	24	**0.014**
Negative microfilaria test^a^	25 (100%)	25	1 (100%)	1	n/a
All-cause mortality^b^	4 (0.8%)	491	4 (2.6%)	154	0.098
Heartworm-related mortality^b^	1 (0.2%)	491	4 (2.6%)	154	**0.013**

Data are shown as median (IQR; range) or *n* (% ± 95% CI). Significant *P* values are shown in bold

^a^5–10 months after the final melarsomine injection

^b^To 8 weeks post-melarsomine

Survival data to 8 weeks post-melarsomine were available for 645 dogs (Table 3). All-cause mortality was 4/491 (0.8%) in the two-dose group and 4/154 (2.6%) in the three-dose group (Fisher’s exact test, two-sided *P* = 0.098; NS). Heartworm-related mortality was 1/491 (0.2%) in the two-dose group and remained at 4/154 (2.6%) in the three-dose group (Fisher’s exact test, two-sided *P* = 0.013). The heartworm-related death in the two-dose group was due to presumed PTE, and those in the three-dose group were caused by an uncharacterized death with no other identified cause, several days after the first melarsomine injection (1); an anaphylactic reaction to the second melarsomine injection (1); euthanasia for severe myelomalacia after the second melarsomine injection (1); and euthanasia for presumed right-sided heart failure several weeks after the third melarsomine injection (1).

Mild post-melarsomine complications/adverse events occurred in 66/889 (7.4%) dogs in the two-dose group and 13/357 (3.6%) in the three-dose group (Fisher’s exact test, two-sided *P* = 0.014) (Table 4). Severe complications occurred in 3/889 (0.3%) dogs in the two-dose group and 5/357 (1.4%) in the three-dose group (Fisher’s exact test, two-sided *P* = 0.048). A breakdown is provided in Table 4.

**Table 4:** Complications and adverse events after melarsomine treatment in 1248 shelter dogs treated with two-dose or three-dose melarsomine

	Melarsomine injection	Two-dose group (*n* = 891)	Three-dose group (*n* = 357)	*P*-value
Total mild	All	66 (7.4%)	13 (3.6%)	**0.014**
Total severe	All	3 (0.3%)	5 (1.4%)	**0.048**
Mild complications/adverse events				
Pain	First	34 (3.8%)	1 (0.3%)	–
	Second	0	1 (0.3%)	–
	Third	–	0	–
Swelling	First	8 (0.9%)	1 (0.3%)	–
	Second	4 (0.4%)	1 (0.3%)	–
	Third	–	0	–
Lethargy	First	5 (0.6%)	0	–
	Second	1 (0.1%)	0	–
	Third	–	0	–
Inappetence	First	16 (1.8%)	0	–
	Second	6 (0.7%)	6 (1.7%)	–
	Third	–	3 (0.8%)	–
GI signs	First	11 (1.2%)	2 (0.6%)	–
	Second	1 (0.1%)	1 (0.3%)	–
	Third	–	0	–
Cough	First	1 (0.1%)	0	–
	Second	3 (0.3%)	0	–
	Third	–	0	–
Severe complications/adverse events				
Anaphylaxis	First	0	0	–
	Second	0	1^a^ (0.3%)	–
	Third	–	0	–
Myelomalacia	First	0	0	–
	Second	0	1^a^ (0.3%)	–
	Third	–	0	–
PTE	First	0	1 (0.3%)	–
	Second	3^b^ (0.3%)	0	–
	Third	–	0	–
RHF	First	0	0	–
	Second	0	0	–
	Third	–	1^a^ (0.3%)	–
Uncharacterized death	First	0	1^a^ (0.3%)	–
	Second	0	0	–
	Third	–	0	–

^a^ Died/euthanized

^b^One dog died

*PTE* pulmonary thromboembolism, *RHF* right-sided heart failure, *GI* gastrointestinal

Data are shown as n (%). Significant *P* values are shown in bold.

Antigen test results were available for 133 dogs that had received ivermectin (*n* = 65), milbemycin (*n* = 35), or slow-release moxidectin (*n* = 33) at intake (administration of a heartworm preventive was recorded for the remainder of the dogs, but the product was not specified). Two dogs that received ivermectin (3.1%) and two that received milbemycin (5.7%) had a positive follow-up antigen test, while there were no positive results for slow-release moxidectin. Almost all of the dogs that received moxidectin (32/33) were in the two-dose group.

## Discussion

The findings of this large retrospective study support our hypothesis that the addition of an ML and doxycycline improves the safety and efficacy of two-dose melarsomine. There was no statistically significant difference between the two-dose and three-dose protocols for NAD, and severe complications and mortality were lower than for three-dose melarsomine. Although mortality in the three-dose group was higher than the two-dose group, this was not considered to be related to the number of melarsomine injections, as two of the deaths occurred on the day of the first injection, two were rare injection complications, and one occurred in a dog that most likely had preexisting right-sided heart dysfunction. The study findings support previous findings that HWD can be safely and effectively treated in shelter and community clinics without routine laboratory or radiographic staging [5, 44].

The efficacy of two-dose melarsomine in this study (96.0%) was not statistically significantly different from that of three-dose melarsomine (100%). In previous field studies that utilized two-dose melarsomine without ML/doxy, NAD ranged from 70.0% to 100% [12, 15, 17, 20, 45]. The efficacy in our study, the first to report on two-dose melarsomine administered with ML/doxy, was higher than most of these studies and was based on a larger sample size than any of them. This supports our hypothesis that the addition of ML/doxy has improved the efficacy of two-dose melarsomine.

NAD for three-dose melarsomine with ML/doxy was 89–100% in previous studies, with the majority reporting 100% efficacy [5, 44, 46–54]. This indicates that it is realistic to expect NAD of close to 100% and that the difference in our study might be clinically important even though it was not statistically significant. However, NAD for two-dose melarsomine in the current study is difficult to interpret because the time to follow-up was significantly shorter than for the three-dose group. Seven of the ten positive tests occurred at 6 months post-treatment, and it is possible that these dogs might have tested negative by 9 months. The discrepancies in sample sizes may also have impacted the results.

Our study findings raise the question of a trade-off between a shorter, less expensive treatment protocol and the possibility of some dogs remaining antigenemic at follow-up testing. Retesting should be performed at 9 months as recommended by the American Heartworm Society (AHS) [2] to avoid unnecessary anxiety, laboratory costs, and premature repeat treatments if the test is positive at 6 months. If the 9-month test is positive, its clinical relevance should be considered. In early melarsomine studies prior to the addition of ML/doxy, 100% of male worms and the majority of female worms were killed by both two-dose and three-dose melarsomine [13]. Considering the independent adulticide activity of MLs and doxycycline [43], the few remaining worms are likely to die prematurely. Persistent antigenemia at 9 months is unlikely to reflect a clinical or transmission risk, given the combined actions of melarsomine, an ML, and doxycycline. A reasonable approach for dogs that are antigenemic at 9 months might be to repeat the test at 12 months before deciding on a further course of action. Another option might be to repeat the course of doxycycline and repeat the antigen test at 12–18 months. Earlier AHS guidelines advised that repeated melarsomine treatment in persistently antigenemic dogs should only be undertaken if there is a strong expectation of additional benefit and that patient age, normal lifespan, health, and performance expectations should be taken into consideration [23, 24]. This remains a reasonable approach.

No previous mortality data have been reported for two-dose melarsomine with ML/doxy. Mortality for two-dose melarsomine was very low and compared favorably with previous studies. Mortality in field studies of two-dose melarsomine without ML/doxy, for dogs with class 1 and 2 HWD, ranged from 0% to 17.4%, with most studies reporting no mortality [12, 16, 17, 49]. In dogs with class 1 and 2 HWD treated with three-dose melarsomine and ML/doxy, mortality ranged from 0% to 3.0% [5, 47, 50, 54].

Complications/adverse events were uncommon in both treatment groups in this study. There is little consistency in reporting and terminology between published studies, for reasons that may include analgesia protocols, case definitions, classification of complications, and the specifics of how complications are monitored. As in previous reports, however, transient injection site reactions were most common, followed by transient nonspecific signs (lethargy, inappetence, gastrointestinal signs) and respiratory complications [5, 12, 16, 17, 22, 32, 44, 47, 50, 55]. Coughing was rarely reported in our study, possibly because it might have been attributed to endemic respiratory infections by shelter staff and was likely to have been managed by technicians without requiring veterinary assessment. Mild, transient complications in owned animals may have been underreported, and some complications might have been managed by private clinics instead of the shelter. Despite these limitations, our data indicate that serious complications were uncommon and that both two-dose and three-dose melarsomine were safe in this setting.

Other than dexamethasone pretreatment prior to preventives in microfilaremic dogs, glucocorticoids were not routinely administered. Glucocorticoids have been administered less consistently than ML/doxy in other studies [47, 48, 51, 54, 56]. In an experimental model, prednisolone decreased the severity of pulmonary lesions associated with implanted dead worms [57], and a 4-week course of prednisone post-melarsomine has been recommended for all dogs since 2018 [58]. Although the potential thromboembolic effects of glucocorticoids have been noted [57–59], the benefits are likely to outweigh the potential risks [59]. However, our findings showed low complications and mortality in a large sample of dogs treated without glucocorticoids post-melarsomine.

While not a primary objective of the study, the choice of ML in association with two-dose melarsomine is of interest. Moxidectin has consistently outperformed ivermectin in non-arsenical adulticide protocols [43], and there is no published evidence to support the use of milbemycin or selamectin in these protocols. We suggest that for the full benefits of adjunctive treatment, ivermectin or moxidectin should be used with the two-dose protocol. All dogs that received moxidectin in our study converted to NAD, and 32/33 of these were in the two-dose group. This suggests that moxidectin could be the preferred ML in the two-dose protocol. This is speculative, and further investigations are needed.

The large sample size was a strength of this study. Owing to its retrospective nature, the study was limited by a lack of standardized reporting and by passive surveillance. Endemic respiratory infections in the shelter may have confounded the attribution of some clinical signs, such as cough, to HW treatment versus canine infectious respiratory disease, resulting in underreporting of HW treatment-associated respiratory complications. Long-term outcomes were unknown for many dogs following adoption or transfer to partner organizations. Despite intensive efforts to obtain follow-up antigen test results, these were found for only 22.2% of dogs and for only 6.7% of the three-dose group. This was due to a combination of factors, including dogs no longer being in the shelter when retesting was due, many having been treated years earlier and low compliance with retesting recommendations following release from the shelter.

The Companion Animal Parasite Council maps over the study period show a gradual decrease in HW prevalence in Miami-Dade County (1.3% in 2014 to 0.8% in 2024) [60]. A smaller reservoir of infection could result in lower worm burdens, which may increase the efficacy of the two-dose protocol [21]. This could also increase the proportion of false-negative antigen tests because the test is less sensitive if only a small number of worms are present [61]. Shelter prevalence and infection intensity data for this period have not been published, and it is not known whether this phenomenon may have influenced the present study findings.

## Conclusions

The study findings suggest that two-dose melarsomine, when combined with an ML and doxycycline, is a potential alternative to three-dose melarsomine in class 1 and 2 HWD. The potential for slightly lower efficacy should be balanced against benefits such as lower treatment cost, a shorter duration of exercise restriction, and, for shelter dogs, a shorter length of stay and removal of an important barrier to adoption. Moxidectin may be a preferred ML in the two-dose melarsomine protocol, but more research is needed to determine this.

## Data Availability

Data supporting the main conclusions of this study are included in the manuscript.

## Abbreviations

HW: Heartworm
HWD: Heartworm disease
MF: Microfilaria
ML: Macrocyclic lactone
ML/doxy: Macrocyclic lactone heartworm preventive and doxycycline
NAD: No antigen detected
PTE: Pulmonary thromboembolism
RHF: Right-sided heart failure
